# Determination of cytokeratins 1, 13 and 14 in oral lichen planus

**DOI:** 10.4317/medoral.19289

**Published:** 2014-03-08

**Authors:** Emilce Rivarola de Gutierrez, Alicia C. Innocenti, María J. Cippitelli, Susana Salomón, Laura M. Vargas-Roig

**Affiliations:** 1 Facultad de Odontología, Universidad Nacional de Cuyo, Mendoza, Argentina; 2 Facultad de Ciencias Médicas, Universidad Nacional de Cuyo, Mendoza, Argentina; 3 Laboratorio de Biología Tumoral- Instituto de Medicina y Biología Experimental de Cuyo (IMBECU) Centro Científico Tecnológico (CCT) CONICET- Mendoza, Argentina

## Abstract

Introduccion: Cytokeratins (CK) are molecules of the cytoskeleton that contribute to the cellular differenciation. We studied the expression of CK1, CK13 and CK14 in thirty-three patients with OLP. The biopsied lesions were located in the dorsal surface of the tongue, the palatal keratinized mucosa and the nonkeratinized buccal mucosa. Objectives: This study aimed to determine the expression of CK1, CK13 and CK14 in oral lichen planus (OLP) and its relations with: clinical patterns, prognosis, drugs and tobacco intake and histopathological features. Study Design: Immunohistochemical analysis, retrospective, descriptive, observational and no randomized study. Results: No significant difference was observed in the expression of CK1 in patients with or without drug treatment. No association was found with the amount of drugs intake or smoking nor with the histopathological features examined. Samples immunostained with CK13 were all positive in the suprabasal layers, and 13 of them in the basal layer. In these last ones, statistical analysis showed significance in the grade of vacuolization of the basal layer (p=0.023) and in the degree of exocytosis (p=0.0025), this, making the degree of affection higher for both parameters. Thirty-two tissue sections were immunostained with CK14. CK14 was expressed in the basal layer in 97% of samples and in the suprabasal layer in 94% of samples. Conclusions: The three CK were altered in OLP. CK1 does not have a direct connection with the presence of orthokeratosis. The finding of the CK13 in the basal layer is related to the agression of the lymphocytic infiltration in the epithelium, due to the basal stratum vacuolization and the increase in lymphocytic exocitosis. The presence of CK14 in the suprabasal stratums is not a parameter to predict malignancy. The CK in OLP do not follow the normal pattern of keratinized or non-keratinized mucosa.

** Key words:**Basal cell vacuolization, CK1, CK13, CK14, cytokeratin, lymphocytic exocytosis, oral lichen planus.

## Introduction

Oral lichen planus (OLP) is a mucocutaneous inflammatory disease which affects 0.5 to 2.2% of population. It usually presents with a chronic course that includes frequent exacerbations (1).

OLP is an example of autoimmunitary damage. The diagnosis must be based on the recognition of clinical alterations as well as on carrying out an interview with the objective of observing a possible cause–effect relationship to differentiate OLP from oral lichenoid reactions (OLR) (2). In this report we only included patients with OLP. It has been reported that the clinical features alone may be sufficiently diagnostic, particularly when presenting in the classic reticular form. The evidence regarding the need and value of biopsy for histological confirmation of the diagnosis is not definitive. Studies have shown variability in both interobserver and intraobserver reliability in the clinicopathological assessment of OLP (2).

Cytokeratins (CK) are a group of the intermediate filament proteins in the epithelium comprising a heterodimer of an acidic and a basic keratin (keratin pair) (3). In 1982, Moll *et al.* found that there were 19 subclasses of CK and classified them according to their molecular weights. CK are place specific and may change when the growth rate rises or when the degree of differentiation is altered pathologically (4). CKs are the main differentiation markers of stratified epithelium.

**Figure 1:**
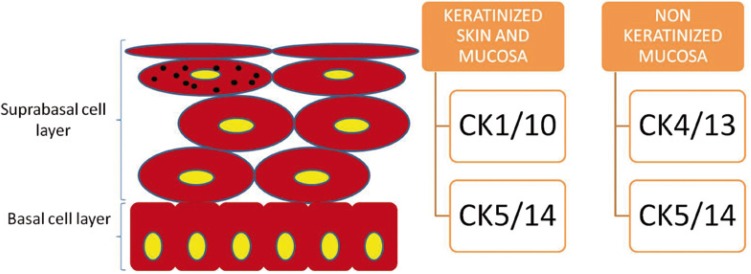
Distribution of CK in normal skin and mucosa.

The pair CK5/CK14 is recognized as a specific marker of the basal layer of normal stratified epithelium (5) and it is the main component of hemidesmosomes (6). As they move away from the basal layer, keratinocytes fail to express these CKs and begin to express tissue-specific CKs. The major keratins of the interfollicular epidermis are CK10 and CK1. However cells of stratified non keratinized mucosa express CK4 and CK13 (7) (Fig. 1).

In oral mucosa, CK4 and CK13 are the predominant suprabasal CKs. However, small subpopulations of suprabasal cells also express CK1 and CK10 (8). Hard palate and gingival mucosa express the same patterns as in the epidermis. The dorsal surface of the tongue and the lateral border contain intermediate patterns between keratinized and non keratinized epithelium (9). In contrast, the alveolar mucosa contains a large proportion of CK4 and CK13 and less CK5, CK6, CK14 and CK17 (10).

CK1 synthesis starts earlier than CK10 which initiates its transcription when there are significant levels of CK1 (11,12) (The presence of CK 1/10 in the epithelium in OLP in the basal and suprabasal layers has been reported) (13).

In this article, we have explored the distribution of CK1, CK13 and CK14 in the oral mucosa lesions in patients with OLP and correlated their expression with clinical and histopathological patient data.

## Material and Methods

Subjects: Thirty female patients and three male patients with OLP, ranging in age from 40 to 85 years (average age, 61.6 years) were included. The biopsied lesions were located in three cases in the dorsal surface of the tongue, in three others in the palatal keratinized mucosa and in the other 27 patients, in the nonkeratinized buccal mucosa ( Table 1). The chief complaint was pain (93.8%). In fact, in the qualitative assessment, through an analogue pain scale from 0-10, patients had an average of 6.45 (SD ± 2.4). In 97.9% of the patients the lesions were multiple, predominantly erosive lesions (n = 37), plaque lesions (n = 20) and ery-thema (n = 12). The main location was in the buccal mucosa (over 70%), followed by the lips, tongue (dorsal and lateral region) and gums. There were lesions in the palate in 12.5% of the participants. The average evolution was 252.7 days with a range of 7-3285 days.

**Table 1:**
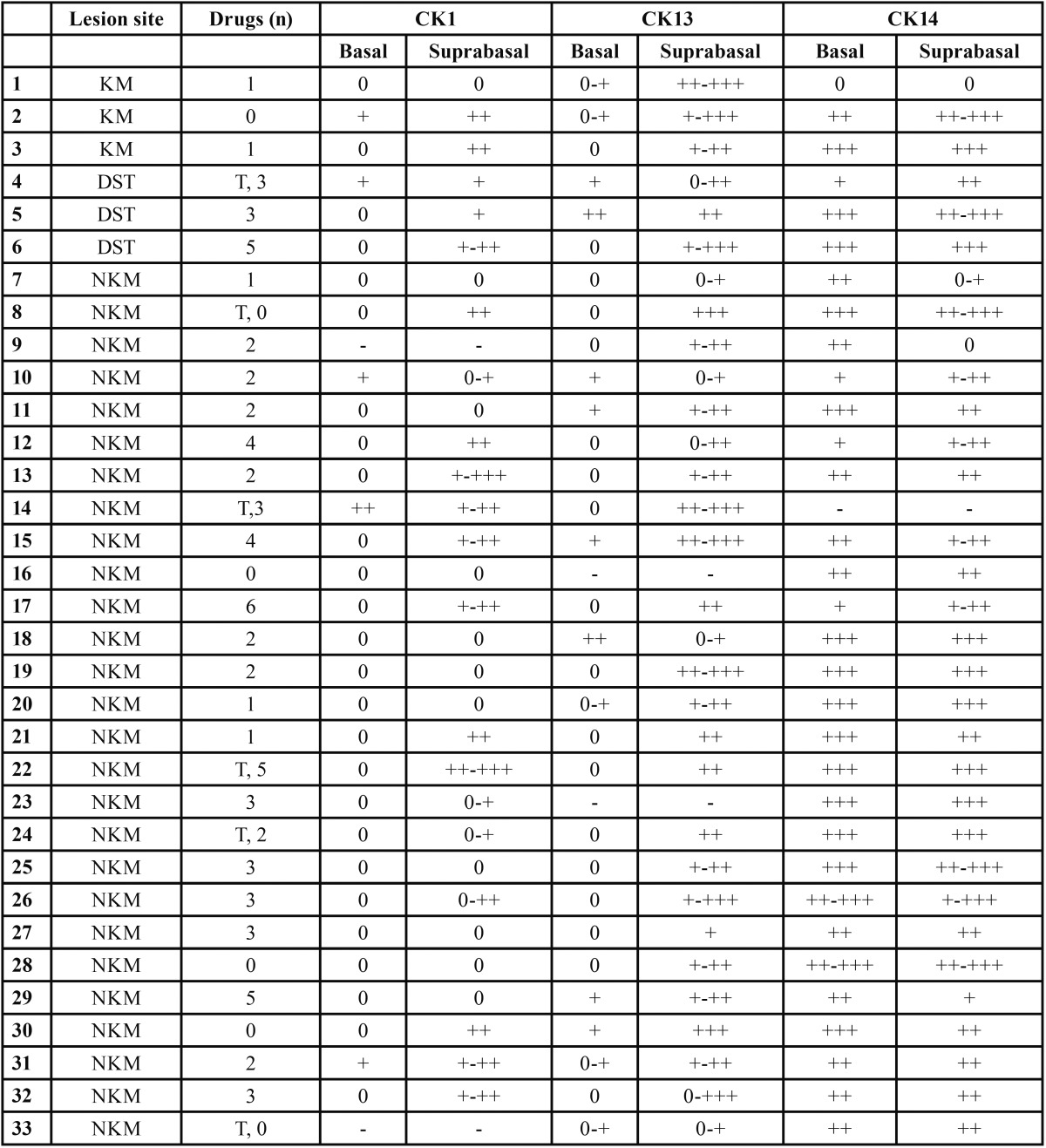
Expression of CK1, K13 and CK14 related to OLP lesions location, tobacco smoke and amount of drugs intake. KM: keratinized mucosa, DST: dorsal surface of tongue, NKM: nonkeratinized mucosa no queratinizada, T: tobacco, 0 No expression, + low expression, ++ moderate expression, +++ intense expression, - CK expression was not obtained due to technical problems.

Inclusion criteria: Biopses obtained from the oral mucosa of patients with clinical manifestations of OLP were included in this study, according to WHO Clinical diagnostic criteria: 1) Presence of bilateral, mostly symmetrical lesions; 2) Presence of network of slightly raised lines (reticular pattern); 3) Erosive, atrophic, bullous, and plaque type lesions in presence of reticular lesions elsewhere in the oral mucosa).

The exclusion criteria were: 1) Patients younger than 16 years old; 2) patients with OLR of graft-versus-host disease; 3) OLR seen in direct topographic relationship to amalgam; 4) OLR in temporal association with the taking of medications, based on the interview and observing possible cause–effect relationship.

Four patients with oral mucocele were enrolled into the study as controls. The present study was approved by the Ethic Committee of the Lagomaggiore Hospital of Mendoza, Argentina. Written informed consent was obtained from each subject. Eighty seven percent of patients had other pathologies and 71% of them were on drugs treatment. The principal drugs administered were: antihypertensives (ACE inhibitors or angiotensin-converting enzyme inhibitors, β-blockers and calcium channel blockers), non steroidal anti-inflammatory drugs (NSAIDs), antidepressants (benzodiacepins), thyroid hormones, calcium and alendronate. Twenty five percent of the patients were tobacco smokers.

Biopsies: Samples were fixed in 10% formalin and embedded in paraffin. Serial 5 µm-thick sections were mounted onto 3-aminopropyltrietoxysilane (Sigma, S. Louis, MO, USA)-coated slides. The histologic specimens were stained with hematoxylin and eosin and the diagnosis was confirmed by optical microscopic examination. The presence of hyperkeratosis, parakeratosis, vacuolization of the basal layer, band-like infiltrate, keratinocytes necrosis, incontinentia pigmenti, dysplasia, lymphocytes exocytosis and sawtooth rete ridges were examined (14). These parameters were graduated: 0= absence, 1= low grade, 2= moderated and 3= severe.

Immunohistochemistry: The immunohistochemical procedure was performed as reported previously (15). Mouse monoclonal antibodies against the CK1 (NCL-CK1, clone 34βB4), CK13 (NCL-CK13, clone KS-1A3) and CK14 (NCL-L-LL002, cloneLL002) (Novocastra, Newcastle Upon Tyne, United Kingdom) were used. The antigen retrieval protocol was carried out in 0.01 M citrate buffer, pH 6.0 at 100 °C for 25 min. Tissue sections were incubated with the primary antibodies overnight at 4°C in a humidity chamber at the following dilutions: CK1, 1:100; CK13, 1:200; CK14, 1:100. As secondary antibody we used anti-mouse IgG biotin conjugate and Avidin and Biotinylated horseradish peroxidase Complex (Vectastain Universal Elite ABC, VECTOR, Burlinghame, CA, USA) were used.

Diaminobenzidine/hydrogen peroxide was used as a chromogen substrate. Slides were counterstained with hematoxylin. The evaluation of the immunostaining was clasified as positive or negative in basal and suprabasal layers.

Statistical Analysis: Fisher’s exact test was used to determine whether the expression of the CK studied correlated significantly with the intake of different drugs or smoke. Mann Whitney test and t test with Welch correction were applied to compare means of the grades of histological changes detected related to the expression of CKs. Statistical analysis was performed using the Prism computer program (Graph Pad Software, San Diego, CA); *p*< 0.05 was considered significant.

## Results

**Figure 2:**
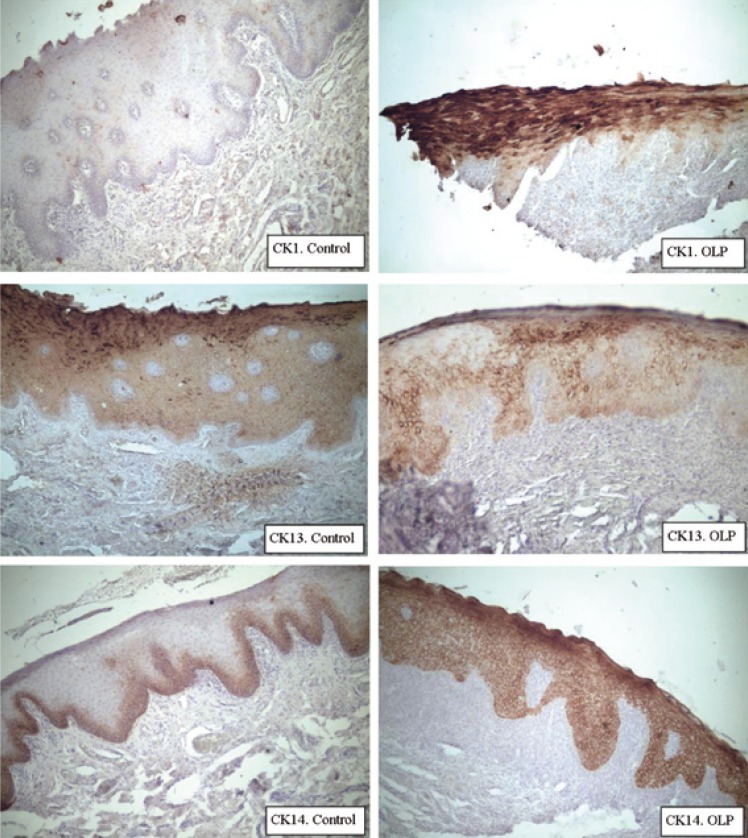
Expression of CK1, CK13 and CK14 in NKM of controls and in NKM of OLP.

In the control group the usual pattern of expression of CK present in non keratinized normal mucosa was observed (Fig. 2). Thirty one biopsies were immunostained with CK1; 5 of them were positive in the basal layer (16%) and 20 were positive in the suprabasal layers (64%) (Fig. 2). In six of these patients, the biopsies were taken from keratinized mucosa (KM); three from hard palate mucosa and three from the dorsal surface of the tongue (DST) ( Table 1). In five of them, CK1 was positive in the suprabasal layer, and in two of those, CK1 was also positive in the basal layer. The expression of CK1 and CK14 was negative in one patient with biopsy from KM and positive for CK13. This suggests a change in the pattern of keratinization. In our patients with OLP, the expression of CK1 in NKM was positive in 15 samples and negative in 10 samples. Tobacco smoke was present in 4 of these patients. CK1 expression was positive in NKM of all the smokers in the suprabasal layers, and also in the basal layer in two of those smokers. In the group of NKM from non smokers patients (n=21) CK1 was positive in the suprabasal layer in 10 patients. No significant difference was observed in the expression of CK1 in patients with or without drug treatment and there was no association with the amount of drugs intake or smoking. When the tisular afectation was compared with the expression of CK1, no correlation was found.

Thirty one samples were immunostained with CK13, resulting all of them positive in the suprabasal layers and 13 positive in the basal layer (42%) (Fig. 2). In KM, CK13 was expressed in 4 out of 6 samples in the basal layer. In NKM, in the basal layer, CK13 was negative in 16 samples and positive in 9. CK13 was positive in the 24 biopsies of NKM in the suprabasal layers.

When CK13 was expressed in the basal cells, statistical analysis showed a significantly higher degree of vacuolization of this layer (*p*=0.023) and of exocytosis (*p*=0.0025) ( Table 2).

**Table 2:**
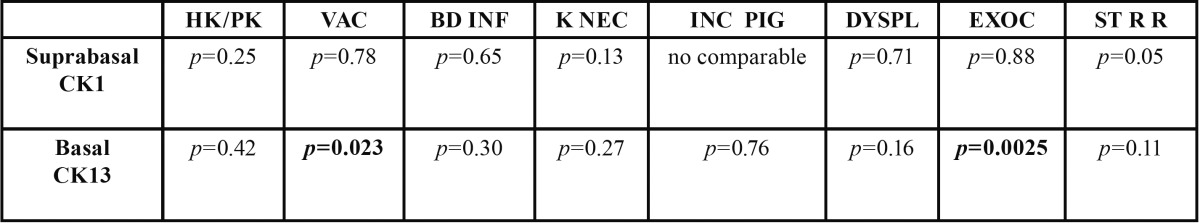
CKs association with histopathological changes. CK1 expressed in 64% of the samples in the basal layer and CK 13 in 42% of the specimen in suprabasal layers. Expression of basal CK1 (16%), suprabasal CK13 (100%), basal CK14 (97%) and suprabasal CK14 (94%) resulted not comparable with histopathological changes. Abbreviations: HK/PK: hyperkeratosis/parakeratosis, VAC: vacuolization of the basal layer, BD INF: band-like infiltrate, K NEC: keratinocytes necrosis, INC PIG: incontinentia pigmenti, DYSPL: dysplasia, EXOC: lymphocytes exocytosis and ST R R: sawtooth rete ridges.

CK14 was expressed in the basal layer of 31 samples (97%, 31/32) and in the suprabasal layer of 30 samples (94%, 30/32) (Fig. 2). The expression of CK14 in the suprabasal layers was more intense than in the basal layer in five samples.

## Discussion

We observed CK1 expression in 64% of our specimens in the suprabasal layer and in 16% in the basal layer. Our research differs from the ones made by other authors, since the presence of CK1 does not have a direct connection with the presence of orthokeratosis. In 1999, Chaiyarit et al., reported that the expression of CK1/10 in the epithelial basal and suprabasal layers was significantly higher in OLP than in the fibroids and that HSP60 presence in the basal layer was significantly superior in the samples of OLP. These authors suggested that many cytokines hidden by the T lymphocytes present during the infiltration, might influence the presence of different CK genes in the adjacent keratinocytes (13).

Later Bloor et al. showed that CK1 and CK10 synthesis was higher in the orthokeratinized epithelium and that the proximity of lymphocytes and their cytokines reduced the presence of the specific CK and their differentiation (8). Van der Velden *et al.* found weak expression of CK10 in OLP and in oral hiperplasias (16) Jaques et al obtained negative CK10 expression in 16/17 samples in the suprabasal layer in OLP and no expression in the basal layer (6). In 2010, Donetti et al proved that the presence of desmoglein 3 (dsg3) and CK10 is altered in the keratinized oral mucosa of smoking patients (17). Chaiyarit *et al* also claimed a hypothesis for the pathogenesis of OLP: in a genetically predisposed individual, an hapten, which is a conventional antigen or a super antigen originated in the oral microbial flora, can lead to an immune reaction, interceded by a cell with a subepithelial infiltration of lymphocytes.

Later, other authors presented similar unifying etiopathogenic models of OLP. At the first stage basal keratinocytes are ‘‘activated’’ by different antigens, which promotes the expression of heat-shock/stress-response proteins in a variant fashion. These proteins may act as a self-antigen induced on basaloid keratinocytes via innate immune response activation. Cytotoxic and pro-apoptotic mediators/stimuli expressed by fully activated cytotoxic CD8 T cells could then mediate the basal cell layer apop-tosis and necrosis that is typical of lichen planus. It is also possible that the first antigen and the second antigen could represent the same molecule (18). An example might be a chemical hapten (19). Some examples of exogenous stimulus capable of starting an autoperpetuating cascade may be certain drugs, dental materials and infections. The generation of cytokines by these cells can positively regulate the presence of HSP60 in the basal adjacent keratinocytes. The next step depends on the individual’s predisposal to a reaction to HSP60. If he is not predisposed, the first immune reaction will result in an unspecified mucositis. However, if the individual is predisposed to react to the HSP60 due to the presence of HLA antigens like the HLA-Bw57 or the HLA-DR2 in his body, then a second immune reaction will continue with the TL development which target the basal keratinocytes, resulting in their destruction (13).

Our findings are greatly supported by this theory, because normally CK1 and CK13 are not expressed in basal layers of the epithelium, neither CK1 in NKM.

In our study, CK13 was observed in 42% of the samples in the basal layer. We found a direct association with the degree of basal layer vacuolization, and the degree of lymphocytes exocytosis with the expression of CK13 in the basal layer. Bloor *et al.* detected CK4 and CK13 homogeneously spread in the suprabasal compartment of the parakeratotic epithelium in OLP (8). The finding of CK13 in the basal layer, suggests a jump in the presence/expression of CK, which should be in superior stratums, and is related to the aggression of the infiltrate to the epithelium, reliable through the basal stratum vacuolization and increased lymphocytic exocitosis.

We observed CK14 expression in 97% of the samples in the basal and in 94% in the suprabasal stratums. The presence of CK14 on the suprabasal stratums may be an indication of a flaw in the cytological differentiation. This may be showing a sort of immaturity in those levels. However, according to our study, this parameter should not be studied since it is expressed in nearly all the OLP, and that is known that not all the OLP suffer from malignant transformation.

It has been reported that patients with OLR have an increased risk of oral cancer, particularly important in patients who have atrophic, erosive or ulcerative lesions (20). As OLR have also clinical criteria, we excluded them from our sample. These include lichenoid contact lesions, lichenoid drug reactions and lichenoid lesions of graft-versus-host disease. In spite of this, in some cases there is a spectrum of OLR that may confuse the differential diagnosis (2). OLP is a chronic disease, in most of our patients it evolved during years. Many of them have multiple amalgam restorations, but at the time of inclusion in this work, they fulfilled the diagnosis criteria for OLP. Besides drug intake was present in most patients, but it had no chronological correlation with the disease.

Our results are concordant with Jaques *et al.* who found CK14 in basal and suprabasal layers in the 23 samples analyzed (6). On the other hand, we do not agree with Brunotto et al, who claimed that the positive immunostaining of CK14 in the superficial epithelial stratums of OLP should be a sufficient malignant sign to begin new exams (5). Our discordance is based on the fact that malignancy of around 5% of OLP has been reported in the literature and that the expression of CK14 is found in 97% of the suprabasal layers. Besides, only one of our patients developed an oral squamous carcinoma in five years follow-up. This patient presented symmetrical lesions of erosive OLP and he was also tobacco smoker. His lesions had clinical WHO criteria for OLP, and he also had typical lichen affection in nails (nail pterygium). Therefore patients diagnosed with OLP must be followed up, as well as those with OLR, especially in erosive, atrophic, bullous and keratotic forms, for early diagnosis of malignant transformation if that occurs (21).

## Conclusions

CK1 was present in 64% of the specimens in the suprabasal layer and in 16% in the basal layer, in patients with OLP.

CK13 was observed in 42% of the samples in the basal layer, this has not been described previously in other studies. The presence of CK13 in the basal layer was associated with a higher degree of vacuolization of the basal layer and with the presence of important exocitosis.

CK14 was positive in 97% of the samples in the basal layer and in 94% in the suprabasal stratums.

The three CK were altered in the OLP. In conclusion, the CKs in the OLP do not follow the normal patterns of keratinization in keratinized or non-keratinized mucous epitheliums.
